# Artificial Intelligence Methods for the Construction and Management of Buildings

**DOI:** 10.3390/s23218740

**Published:** 2023-10-26

**Authors:** Svetlana Ivanova, Aleksandr Kuznetsov, Roman Zverev, Artem Rada

**Affiliations:** 1 Natural Nutraceutical Biotesting Laboratory, Kemerovo State University, Krasnaya Street 6, 650043 Kemerovo, Russia; 2 Department of TNSMD Theory and Methods, Kemerovo State University, Krasnaya Street 6, 650043 Kemerovo, Russia; 3 Computer Engineering Center, Digital Institute, Kemerovo State University, Krasnaya Street 6, 650043 Kemerovo, Russia; adkuz@inbox.ru; 4 Digital Institute, Kemerovo State University, Krasnaya Street 6, 650043 Kemerovo, Russia; r.zverev@i-digit.ru (R.Z.); rada.ao@kemsu.ru (A.R.)

**Keywords:** artificial intelligence, construction objects, digital twins

## Abstract

Artificial intelligence covers a variety of methods and disciplines including vision, perception, speech and dialogue, decision making and planning, problem solving, robotics and other applications in which self-learning is possible. The aim of this work was to study the possibilities of using AI algorithms at various stages of construction to ensure the safety of the process. The objects of this research were scientific publications about the use of artificial intelligence in construction and ways to optimize this process. To search for information, Scopus and Web of Science databases were used for the period from the early 1990s (the appearance of the first publication on the topic) until the end of 2022. Generalization was the main method. It has been established that artificial intelligence is a set of technologies and methods used to complement traditional human qualities, such as intelligence as well as analytical and other abilities. The use of 3D modeling for the design of buildings, machine learning for the conceptualization of design in 3D, computer vision, planning for the effective use of construction equipment, artificial intelligence and artificial superintelligence have been studied. It is proven that automatic programming for natural language processing, knowledge-based systems, robots, building maintenance, adaptive strategies, adaptive programming, genetic algorithms and the use of unmanned aircraft systems allow an evaluation of the use of artificial intelligence in construction. The prospects of using AI in construction are shown.

## 1. Introduction

Many unpredictable factors, including the human factor and the unstable environment in conditions of great uncertainty, make the building sector one of the riskiest. Most countries experience high rates of urban and industrial development, and therefore it is difficult to prevent a high level of mutilation and accidents on building sites. Falling from a height, blows, abrasions and electric shock are among the most common kinds of accidents [1]. The level of fatal accidents in the building sector is higher than in other areas and accounts for 30.44–40.06% of deaths worldwide [2]. The rate of non-fatal mutilation in the building sector is 71.51% higher than in other economic sectors [3]. Among the main factors affecting the level of injuries, the absence or insufficient training of employees, poor awareness of the dangers by project managers and reckless actions of employees should be noted [3]. Artificial intelligence (AI) is the capability of a computer to learn, make decisions and perform actions characteristic of human intelligence [4]. In general, AI combines mathematics, computer science, biology, psychology, cybernetics and many other sciences. AI technologies allow for the creation of algorithms and programs that train computers to solve problems independently.

Approximately 7.22% of the world’s working population are employed in the construction sector. Despite its economic importance, the obvious problem of this area is low labor productivity in the construction process, which negatively leads to the waste of labor, material resources and financial resources. Since construction activity makes a great economic contribution to the development of society, it makes sense to carry out proper construction management in order to raise productivity. If construction productivity increases by 50.11–60.04% or higher, it is estimated that this will bring an additional RUB 1.6 trillion in profits to the industry each year and further increase global GDP [2].

**Figure 1:**
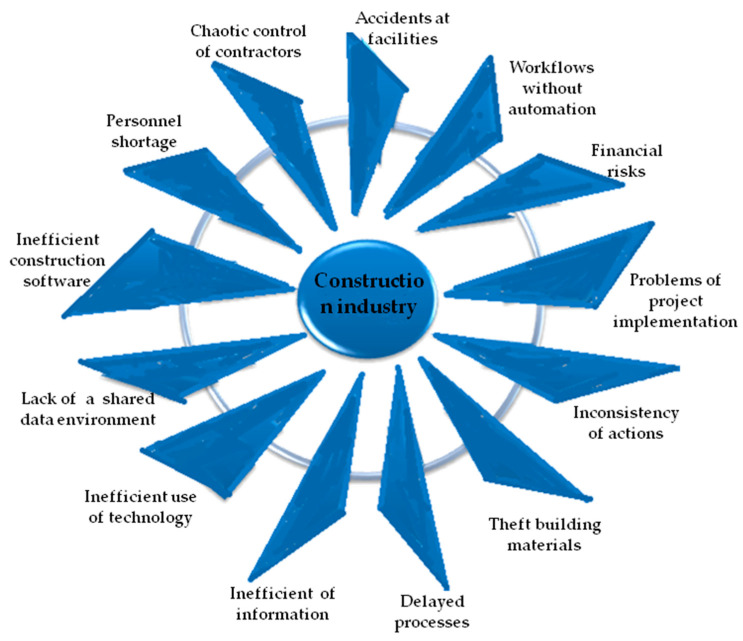
Key problems of the modern construction industry.

One of the restrictions of the industry’s development is low digitalization and the predominance of manual labor. This leads to the inefficient use of funds and resources, project delays, poor quality of execution, low performance indicators and risks to the health and safety of employees [5]. The construction industry faces many problems (Figure 1) that hinder its growth and lead to an extremely low level of productivity compared to the manufacturing industry [6]. Indeed, the building sector is one of the least digitized branches of production in the world [7]. The lack of digital technologies and the excessively manual nature of the branch makes project management unnecessarily wearisome and more complex [8,9]. The lack of adequate digital knowledge and technology implementation in the building branch is associated with project delays, cost inefficiency, poor quality of execution, uninformed decision making and low productivity as well as health and safety indicators [4]. Taking into account the existing problems of labor shortage, the need to ensure sustainable construction rates, the COVID-19 pandemic and global financial crises, it became obvious that in the coming years the construction industry should rapidly develop its technological potential and intensively implement AI methods [10,11,12,13].

AI in construction refers to the use of computer systems and algorithms to perform complex construction and construction management tasks [2,4]. It is expected that AI technologies will determine the development potential of the building branch in the coming years.

Over the past ten years, studies show that the application of AI technologies is increasingly spreading across the building sector: from all areas of architectural and engineering design to building services [14], with artificial neural networks (ANN) replacing building professionals such as architects. The first such developments in the construction industry emerged in the 1960s, for example, an architectural machine [15]. Over the past half century, many works have been published with solutions to specific construction tasks using various artificial intelligence technologies. Thus, machine learning (ML) [16] has replaced knowledge-based systems as a new area of interest for the building branch, while optimization [13], robotics [17] and 3D printing have become the most interesting topics of research on the use of AI in construction. In the second decade of the 21st century, various digital technologies, including augmented reality (AR), blockchain, Internet of Things (IoT) and quantum computing, were integrated with AI to solve problems in the building industry [18,19]. In recent years, AI research in the building sector has focused on the development and implementation of various methods: the probabilistic, statistical and economic methods, including formal logic, mathematical optimization, artificial neural networks, received the combined name of architectural artificial intelligence (AAI). The development of the building branch was affected by the complexity of the technologies used [20].

Industrialization, digitalization and globalization have significantly influenced the changes in the building branch. There is an opinion that the next major changes that the industry will face over the next five years will include consolidation, customer focus, human resources, internationalization, investment in technology, product approach, specialization, sustainability value chain and supply chain management [21]. The current shift from project-based building processes to a product-based approach will change these innovations. Companies will produce structures outside the construction site, instead of on-site construction similar to the processes in other types of production activities [7]. It is expected that in the future, the construction process will be more product-based, so the design and products will be produced outside the construction site. Moreover, the degree of internationalization will be increased by consolidating the value chain [22].

Analytics and customer behavior data will be used to optimize future projects. The degree of development of AI technologies will determine the future of the design, ecosystem or schedule of building, workplace productivity and occupational safety. Digitalization is necessary for data-dependent decision making and consolidation of the value chain [23,24].

The effectiveness of artificial intelligence and machine learning in the field of implementation reaches robotic bricklaying, welding, pouring concrete and even erecting the entire building using 3D printing technology.

Such software as Allplan (starting from version 2014), ArchiCAD (starting from version 2.0), AutoCAD (starting from version 7.1), nanoCAD (starting from version 3.0), Revit (starting from version 8.0), SCADOffice (starting from version 11.5), Compass (starting from version 5.0), etc. are particularly popular in construction [25]. The AutoCAD program allows for the creation of necessary drawing documentation and 2- and 3-dimensional models of the projected object using the built-in library of graphic elements. There are printing control capabilities even for three-dimensional objects [25]. The advanced capabilities of AutoCAD are concentrated in the following applications: Architecture (documents and drawings); Civil 3D (infrastructure design, land management, landscape, etc.); Inventor 3D (design of complex sites); and Navisworks (monitoring of architectural projects). Commercial use of AutoCAD requires the purchase of a license [26]. ArchiCAD is considered one of the best computer applications for architectural and construction design (creation of virtual models of building construction). It is also possible to create project documentation for the object being developed [27].

The scientific novelty of the study is presented in a review of modern literature concerning the issues of using AI. For the first time, published research data on the use of AI in automating routine but important tasks for construction management, for competent task planning, to prevent component delays, to resolve various kinds of conflicts and to meet deadlines for the completion of objects are analyzed. For the first time, arguments are presented that, by using AI, organizations can identify and eliminate potential problems faster than with the help of human potential. It has been found that project managers prefer to use AI to verify and analyze significant amounts of data.

In our opinion, the specifics of AI in construction are as follows: Firstly, the duration of the assessment of the real effectiveness of innovations associated with the life of the building, during which certain shortcomings of the design and technological solution can be identified. In this regard, it is necessary to apply balanced and reasonable approaches to the selection of innovative building materials and technologies in the construction of real estate. Secondly, the high level of responsibility of builders for the final result and the consecutive manifestation of a certain conservatism in the use of AI in order to eliminate errors in the design or in the choice of technologies, as well as to reduce the level of risk to human life and health. Thirdly, the formation of certain stereotypes and traditions as a result of a long history of AI development in construction, and the consecutive use of tested materials, as well as design and technological solutions that are quite difficult to modify [23].

Therefore, achievements in the sphere of AI and machine learning have also touched on the main problems of the building branch. The aim of this work was to study the possibilities of using AI algorithms at various stages of building, including ensuring the safety of the process. The main sections of this article are the history of creation, relevance, problems and prospects of using AI in the construction industry. The collected information can be useful to both researchers and specialists engaged in the building branch.

The main issues to study in the article are the following. This study describes the following types of AI: 3D modeling for building design, BIM uses machine learning to conceptualize design areas in 3D, computer vision, ALICE—construction planning, image recognition technology for tracking working hours, machine sensors—planning the effective use of construction equipment, artificial narrow intelligence (ANI), artificial general intelligence (AGI) and artificial superintelligence (ASI). It also describes automatic programming for natural language processing, knowledge-based systems, robots—design, creation, operation, maintenance of buildings, adaptive strategies (ES), adaptive programming (EP), genetic algorithms (GA), differential evolution (DE) and particle swarm optimization (PSO) mapping using unmanned aerial systems. Our review is not systematic. The authors are aware that it is impossible to cover the entire volume of materials on the presented topic, and conclusions were drawn only from the information provided by the reviewed works.

The main hypothesis of this work is the justification of the necessity and acceptability of the use of AI in construction and other industries. It is planned to conclude that the use of AI, innovations and nanotechnology improve the quality of the finished product and reduce construction time and costs. There are a lot of studies in the literature on the use of AI in science and technology [7,21,22,23,24,25,26]; however, there is very little full-fledged information about the use of AI in construction [23].

**Figure 2:**
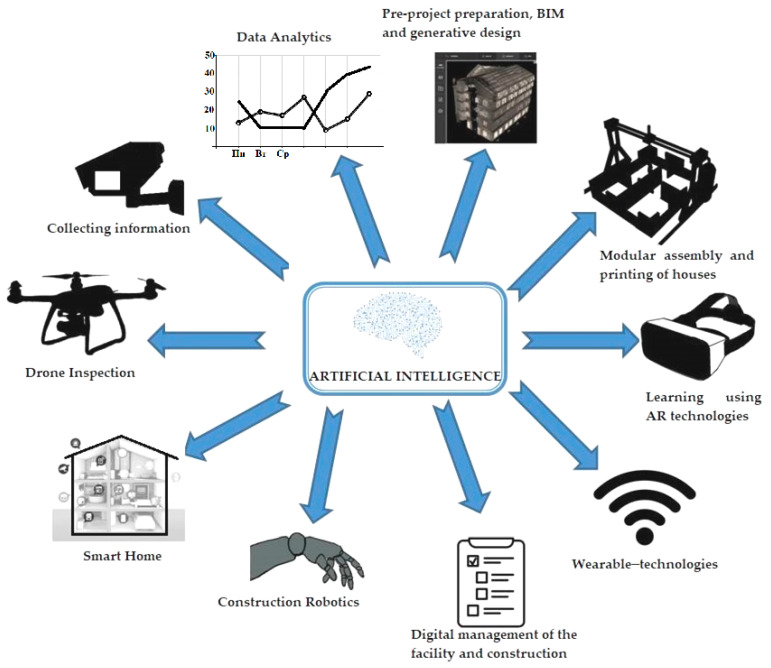
Types of AI and their use.

The scientific novelty of the research lies in the systematization of the existing global experience in the use of AI at all stages of the construction process as well as in the development of the conceptual and categorical apparatus of AI in the construction sector. A diagram that provides a detailed view related to the implementation of a generalizing literature review is presented in Figure 2.

The construction industry is not well-receptive to innovative technologies. This is manifested in the low level of costs for research and development work, the conservatism of design and contracting organizations, as well as the traditionalism of controlling state bodies. However, in the last decade, the process of introducing innovations into the investment and construction sector has noticeably accelerated. Computer methods of modeling all stages of the production and construction cycle have become widely implemented [22]. A serious transformation of raw materials, materials and construction technologies used in the industry is predicted, which entails an inevitable organizational revolution in the construction industry and the integration of interaction between architects, designers, construction and maintenance services [23].

It should be noted that the pace of scientific and technological progress in construction is closely related to the speed of transition to innovative, automated methods of building construction facilities, robotic complexes and technologies with minimal human labor. Today, many experts agree that one of the promising trends in the construction industry is an accelerated transition to the model of modular housing construction, and, consequently, to the conveyor production of real estate objects using unified panel or modular elements and AI algorithms [21].

The unique contribution of this study to the use of AI is as follows. For the first time, a study of all types of world literature on the generalization and search for ways to improve the quality of construction using innovative technologies and artificial intelligence was conducted.

## 2. Methodology

The objects of this research were scientific publications and patents of Russian and foreign authors concerning the use of artificial intelligence in construction and ways to optimize this process. To search for information, Scopus, Web of Science, and Elibrary databases were used for the period from the early 1990s (the appearance of the first publication on the topic) until the end of 2022. The available review and research articles in English and Russian on 3D modeling for building design, the use of machine learning to conceptualize design areas in 3D, computer vision, image recognition technology for tracking working hours, planning the effective use of construction equipment, artificial narrow intelligence, artificial general intelligence, and individual articles related to the justification of the relevance of the topic, understanding the properties and mechanisms of the use of artificial intelligence in construction, and identifying promising areas of research in this area were selected and analyzed. The main focus was on articles published in scientific, peer-reviewed journals with a high citation index over the past five years. Conference materials and book chapters were also used in the analysis. At the same time, articles available only in the form of abstracts, as well as bibliographies, editorial materials and articles published not in English were excluded. Generalization was the main method. Statistical and research data related to the study of the use of automatic programming for natural language processing, knowledge-based systems, robots, their design, creation and operation were analyzed. The authors considered arguments based on the hypotheses of leading scientists about the influence of artificial intelligence in construction and its optimization and formed their own opinion based on the evidence of these hypotheses.

## 3. History of Creation and Management of Smart Objects Using Artificial Intelligence

Humanity has been preoccupied with the idea of creating artificial intelligence throughout its existence, and the active implementation stage was launched with the invention of computers [28]. The term “Artificial Intelligence” was introduced in 1956 by John McCarthy at a scientific conference on this topic at Dartmouth College [29], but there is still no single definition of this concept. Artificial intelligence can be understood as a set of algorithms and system products capable of performing actions characteristic of human intelligence. The capabilities of AI and the breadth of its application are limited only by existing computing power.

It is believed that AI can provide businesses with new economic benefits. At the moment, innovative technologies are the defining resource and the main factor in the modernization of the economy of any country. Therefore, the possibilities of using machine learning, computer vision, and neural networks in various spheres of the economy attract the attention of researchers and manufacturers around the world [30,31,32,33,34,35,36,37,38,39,40,41,42,43,44,45,46,47,48,49]. The construction industry is no exception. The history of the creation and management of smart objects using artificial intelligence technologies in the industry is closely related to robotics [50].

Robots have significant potential for use on the building site. The areas of the building branch in which robotics can be applied are described in [51]. Based on the performance requirements, a conceptual description of four types of construction robots has been prepared. The effectiveness of the use of these robots is noted, indicating the presence of possible specific problems. The authors [52] believe that most of the construction tasks can be performed by a robot assembler (moving large building components), a general-purpose robot (interior finishing work, exterior walls, etc.) and a robot finisher (finishing large vertical and horizontal surfaces). The study shows the economic efficiency of these robots due to stable performance and quality of work compared to humans. The study [53] describes a robotic arc welding system with numerical control, which was developed using artificial intelligence technologies. The system includes three types of robots and complex software for preparing and controlling the welding process. The study [50] describes a method for finding the optimal number of workers with equal functions performing joint work on construction sites.

The potential of automated data collection In real time, process control and robotics for remote, large-scale engineering building projects is presented in [51]. Classifications of automation technologies and robotics in such operations are based on hardware, remote sensing, analog and digital telecommunications technologies, optical data transmission methods, monitoring by computer control of devices, automatic control of mobile equipment, stationary manipulators, mobile robots, etc. The addition of selected technologies with appropriate software increases the efficiency of processes, facilitating data analysis and design and making management decisions.

The combination of artificial intelligence methods and expert systems expands the possibilities of reproducing human experience and expert activity. A fundamental limitation of traditional planning methods is their ability not to use the information obtained when creating a project plan but only the data generated during planning [52]. Artificial intelligence technologies provide opportunities to create plans, substantiate stored information and draw conclusions based on them. The use of AI of a limited amount of subject domain information for planning, leads to inadequate solutions to complex real-world project planning tasks. Construction planning includes the choice of construction technology, the definition of work tasks, the assessment of required resources and duration, and cost estimates. In the development of planning systems the methodology of receiving information is used. The 1985 study [53] describes the areas of application of expert systems based on information in the area of the control and monitoring of building projects. The developed expert systems are computer programs that can replace highly qualified specialists. The ability of these systems to solve poorly structured problems and to modify the solutions found makes their application in this field desirable. Several expert systems used at different stages of development in construction engineering both for assessing the safety of buildings during construction and for choosing methods for the construction of bridge buildings were discussed in [54]. The 1987 review [55] notes that there are many different expert systems that differ conceptually and by means of implementation, which have the potential to be used in construction projects. A prototype of the expert system using the INSIGHT program (2 microcomputer expert-system shell) was developed for the construction of precast, reinforced concrete constructions [56]. The criteria were collected from experienced construction experts in this area. The system recommends the numerical size of the construction team to perform the main work on the construction site, determines the amount of expected productivity and formulates production problems affecting productivity.

Saleh [57] presented a computer program that automates the preparation of a project document and complements the standard AutoCAD system for extracting project data necessary to perform an automated quantitative statement. The program is written in C and, if necessary, can interact with other databases that the project designer or contractor works with. The availability of an accurate and automated method for compiling a statement of work volumes from such libraries creates a benchmark in professional construction practice. This study presents a computer program developed by the author. This program is a third-party achievement that automates the above-mentioned task and complements the standard AutoCAD system, which is not able to extract project data related to the implementation of an automated statement of work volumes. The program is written in C and has the ability to interact with other databases owned by the designer or contractor. AutoCAD was chosen as the base software because it is a cost-effective CAD software, although Archicad, Design + available software can perform automatic quantity analysis [57]. In [58], a forecast of the quality level of concrete constructions is carried out using the QL-CONST1 expert system, which is able to analyze information generated by human experts in a specific field of construction engineering and structural safety. The system of automatic design of wooden frame houses was developed to perform routine operations (accurately and quickly builds drawings and forms estimates) using AI technologies [59]. In [60], two planning systems—LESP 2 (a training system for drawing up an inspection plan) and IDA (a system for finding solutions in construction)—are analyzed.

Deterministic recommendations of professional consultants on the cost of construction can lead to inadequate investment decisions of developers and investors in the construction sector. In particular, expert systems [61] based on probabilistic methods and other forms of AI make it possible to clearly take into account uncertainty and incomplete information, and the contribution of these methods to risk assessment leads to more realistic investment decisions in conditions of uncertainty or incomplete information. The prototype expert system CONSTRUCTION PLANEX [62] is designed for planning modular, high-rise buildings, including the excavation, foundation and construction of buildings, generates networks of design work, cost estimates and schedules, including a description of activities, specification of precedents, selection of suitable technologies and assessment of duration and costs.

Predicting activity on construction sites using AI is a powerful tool because it allows for the provision of detailed information about current performance, the need to increase speed and also helps to reduce the risks of future actions. In parallel, a huge number of scenarios are simulated, which allows for the quick prediction of probable events and the ability to “win back” one’s main actions in the real world without taking any real risks [63,64]. The introduction of AI-based expert systems into construction takes place in several stages [65]. Information about the object necessary for analysis is entered into the data processing program. Increasing the amount of initial data collected reduces the number of incorrect conclusions. Many construction companies already use automated systems that collect the necessary data, or data from different sources are combined, which requires more time and effort [65]. Next, a new algorithm is created or an existing one is being refined [66]. At the last stage, the algorithm is trained and/or self-taught in order to create a comprehensive strategy for the logistics of construction and all relevant business processes of the construction company, taking the AI capabilities into account [66].

## 4. The Role of AI in the Building and Management of Smart Objects

More and more AI is turning into an autonomous, smart assistant, which is able to emphasize the advantages of the construction business in such matters as budget management, financing, drawing, management of automated devices and mechanisms, safety and accuracy of production, project planning, communications management, etc. [67]. Figure 2 shows the types of AI and their use.

Investors around the world spend more than RUB 10 trillion a year on construction-related activities, and this figure is expected to grow by another 42.36% by the end of 2023 [4]. The McKinsey report for 2020 [66] noted a lot of work on applied construction solutions that introduce artificial intelligence into the development of house projects and building. According to the report [67], as a new technology, artificial intelligence in construction will approach RUB 4.5 billion by 2026. Artificial intelligence tools perform complex and expensive tasks more accurately than humans. Over the past decades, AI has proven to be a fantastic tool in the building sector, allowing for the conservation of money and time and the creation of incredibly beautiful houses and buildings around the world [68]. In the nearest future, artificial intelligence and incredible computer-generated ideas will displace building engineers and architects [69]. The drone hovers over the construction site and almost immediately transmits observations to a tablet or computer (e.g., violations of safety by the workers, swampiness of the site, insufficient use of construction equipment, etc.) [70].

The ML construction program [71] predicts possible risks during construction using the initial data before the building. The developed technology saves the time and work of the expert team at the design stages and time-consuming planning stages.

In commercial organizations that use sensors for data collection and IoT in the building, some success has been achieved in studying performance and resource consumption [72]. The neural system is formed from small sensors mounted on the ceiling. After the collected information is sent to the cloud by platforms such as MindSphere, Siemens or Schneider Electric EcoStruxure, the data obtained are interpreted and help engineers control the costs and use of utilities [73]. Companies producing synthetic construction management software (PlanGrid 2023 or Procore 2023) are constantly improving the technology of using machine learning in their processes [74]. These technologies help prioritize equipment maintenance tasks and reduce the risks of injury at work. AI methods in construction develop management of a developing building in the shortest possible time, reducing the need for an architect to design, a bank that pays for the development of the project and a manager who controls all construction functions.

In studies [75,76], AI technologies were used in the construction of railway stations and bridges. Most of the existing railway stations in the UK are historical buildings and have been in operation for many years. The digital twin or building information modeling (BIM) is applied to the King’s Cross Railway Station building in London using Revit-based building modeling. Implementation and transformation of a 3D model of the station building into a 6D information model of the building contained characteristics of time, costs with the calculation of carbon emissions and assumptions about reconstruction using Revit. The study [75] presents reasonable recommendations for the implementation of BIM in railway station projects, which can be useful in the design, planning, and operation of both environmentally friendly and financially profitable construction projects.

## 5. The Relevance of the Use of AI in the Building Sector

The main AI methods [4] used for the smart construction of buildings and their management are machine learning, computer vision, robotics, automated planning, knowledge-based systems, optimization, etc. (Table 1).

Eastman was the first to create the concept of a computerized building design system with all the functions of modern building information modeling [119]. The basic functions of building information modeling took a quarter of a century to enter the market, while some, such as automatic verification of building codes in design, have yet to be fully implemented. Warszawski and Sangrey pointed out in 1985 that “Robotics can be introduced in construction in several ways: a simple development of robotics and computer technology within existing procedures or an impressive combination of robotics and computer-aided design/automated manufacturing, defining the basis for completely new construction systems (construction of the future) [50].

Various methods of artificial intelligence, the use of which will lead to a more efficient construction process (automated, reliable and self-modifying, thus, saving time and finances) are of great importance for the development of the construction industry [2]. Many artificial intelligence technologies are new, although their ideas in the field of construction were formed several decades ago but have not found practical application due to the lack of a reliable digital information base of buildings. The idea of building information modeling was first mentioned in a 1975 article [119]; the ideas of using AI tools and code verification appeared in the mid-1980s [120].

There is a construction tech application. It is based on the building information modeling and offers the following digitalization tools [120]:

- Software tools that manage design and construction;
- Software and hardware complexes for delivering information from project to object (BIM-to-field); this group includes tools that through users’ mobile devices deliver information about products and processes directly to workplaces in the field;
- Robotic applications for performing construction work on the site;
- Software and hardware complexes for collecting information from an object and providing it to control functions (field-to-BIM).

The introduction of artificial intelligence technologies in the construction sector is currently in its early stages but has great potential for the industry’s transformation.

## 6. AI to Ensure the Safety of Construction

**Figure 3:**
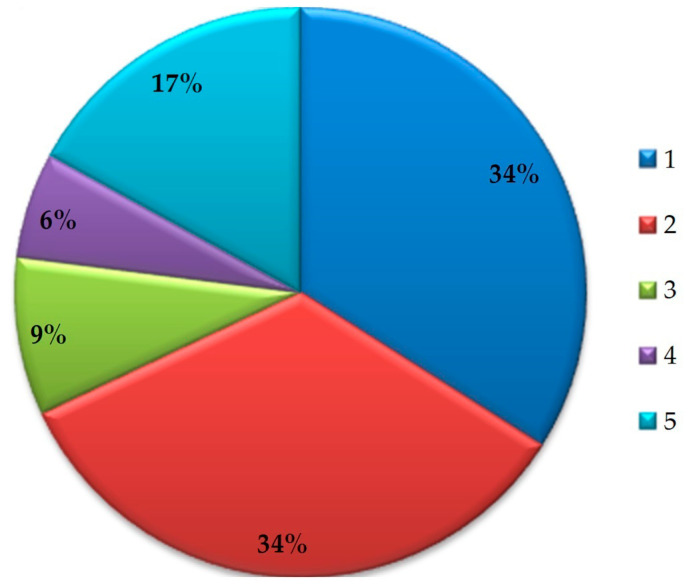
Statistics of accidents in the building sector: 1—falls from a height; 2—bruises; 3—electric shock; 4—entrapment; and 5—the rest.

Accidents often occur during construction, which shows insufficient attention to safety issues, a lack of effective safety supervision and a lack of safety knowledge among construction personnel [121]. Statistics of accidents [122] in construction are presented (Figure 3). In 2020, 1008 deaths were registered at the construction site. A construction worker has a 1/200 risk of getting fatally injured throughout their career [123]. About 60.41% of construction accidents occur during the first year of work at the construction site [122]. About 2.3 million working days are missed per year due to construction accidents [124].

To improve building processes and the level of personnel safety, new technologies are constantly being introduced into the construction sector (Table 2). Zhang has created a real-time accident detection and early warning platform. As a result, warnings are sent automatically when a risk occurs. Experimental results show that when using artificial intelligence technology and computer vision to manage the safety of civil construction, the safety management of workers in construction is implemented, and the level of the construction safety management has increased to 97.41% [121]. Bigham et al. have developed an AI platform that enhances construction safety by identifying hazards at the pre-construction stage and offers applicable Occupational Safety Management codes for the identified hazards. The authors pay special attention to the main cause of building accidents (the risk of falling from elevations) [125]. In recent years, the development of technologies and appropriate software for machine learning has been aimed at providing improved solutions for potential security threats and risks in the construction environment [126].

Huang et al. [135] presented new concepts for the use of AI in building fire safety for predicting dangerous situations: AI and Big Data for predicting limiting design conditions to reduce the cost of fire protection systems; digital twins for fire extinguishing in a building, combining a network of IoT sensors to visualize smoke in buildings in real time; and Digital twins and a real-time AI-based fire prediction mechanism.

In megaprojects, accounting systems are becoming increasingly important when ensuring security. The collected data is transferred to large repositories of information, as there is a need to investigate problems to the smallest detail in order to solve them correctly. The collected data are characterized by a significant level of heterogeneity due to the large number of characteristics and types of information. For the virtual presentation of the behavior of objects in construction, the digital twin technology appears promising. This makes it possible for the operator to control the worker and equipment actions to prevent accidents [136].

To predict construction accidents, Ayhan and Tokdemir [127] used two technologies: an artificial neural network and case-based reasoning. The artificial neural network’s performance was worse than the case-based reasoning’s. In test cases with a fatal outcome, the prediction accuracy of case-based reasoning was 86.31%, and the prediction error did not exceed 18.23%. The researchers presented preventive actions to eliminate safety violations that should be implemented before and during the construction phase. To study the normal functioning of the system Gheraibia et al. [128] proposed a new approach using machine learning in combination with real-time operational data. Subsequently, if there is any abnormal situation regarding the normal functioning model, the method allows for the ability to find an explanation for the anomaly in the failure tree and then share this information with the operator. If the failure tree cannot find an explanation for the situation, a number of different recommendations including potential adjustments to the failure tree are provided depending on the nature of the situation. When creating a road construction safety monitoring platform, Zhu et al. [137] combined UAVs and artificial intelligence technologies. To compensate for the lack of surveillance data from the road construction site, the proposed technology made it possible to control the safety factors of road construction in general: for builders, for construction equipment and safety signs, and fences. For automatic detection and tracking of security factors, deep learning algorithms included YOLOv4 and DeepSORT. Despite the fact that the proposed platform demonstrated good results in field trials, this study had some limitations. Firstly, the characteristics of the monitoring platform applying to an unmanned aerial vehicle (UAV) were determined by the stability, durability and other parameters of the UAV itself. Secondly, there was a need to increase the efficiency of the UAV object detection algorithm due to the uniqueness of the survey. In order to use the proposed monitoring platform in different regions, it is necessary to ensure that all the requirements and laws of UAV operation are met for different methods of controlling its flights.

Yeon-Chul et al. [129] applied artificial neural networks to predict accidents during construction and to assess its applicability. As a result, the ANN model showed higher prediction accuracy (80.16%) than discriminant analysis models (70.11%). It is expected that the presented model can be used as a practical guide for the prevention of accidents on construction sites. Li et al. [130] described the method of rapid object detection by regional convolutional neural networks to detect the danger of construction on objects and use mixed reality so that artificial intelligence could detect danger. The rapid detection of objects by a regional convolutional neural network allows for the acquisition of expert knowledge for the identification of objects in the image. Unlike image classification, the complexity of object detection always implies an increase in complexity, which requires solutions in terms of speed, accuracy and simplicity.

The process of ensuring safety at the construction site should begin from the very planning stage. BIM can also help improve safety planning at construction sites. BIM helps in verifying the detection of incidents that may occur during construction, and many security threats can be avoided by planning in advance. Since manual inspection can lead to some errors, detecting the behavior of workers in real time can help reduce the number of accidents on construction sites. With the help of AI, one can easily monitor the safety of construction sites. Computer vision was used to develop a safety model. By training the model using a quantum number of images, the proposed model helps to assess safe and unsafe conditions on construction sites and, thus, to a certain extent, indirectly reduce the number of accidents at construction sites [138]. 

Based on the data (2011–2016) on industrial accidents from the Ministry of Employment and Labor Relations of the Republic of Korea, Choi et al. [131] developed a model using machine learning to predict the potential risk of fatal accidents at construction sites. The data presented contain information on 137,323 injuries and 2846 deaths (date of the accident, employer’s scale, age, gender and work experience of each victim, and type of construction). AI methods (machine learning, logistic regression, decision tree, random forest and AdaBoost analysis) were used to identify the distribution of a dataset with the allocation of significant variables affecting the classification in each algorithm. The prediction accuracy of the presented model was quite high (91.98%). In this study, random forest analysis identified significant factors (in descending order of significance): month/season, employment level, age, day of the week and work experience.

Helmets play an important role in protecting builders from accidents. However, the wearing of helmets among workers is not strictly observed for various reasons. In order to increase the safety of construction sites, in most ongoing works, the availability and proper use of protective helmets are monitored by multi-stage data processing with restrictions on generalization and refinement. A convolutional neural network is the basis of a one-step system that automatically tracks the presence of protective helmets on construction personnel, determining the appropriate colors. The accuracy of the evaluation by the developed system is 83.89% [132].

The study [20] presents quantitative models for predicting types of injuries (ML algorithms, including fine trees, ensemble of reinforced trees, xgboost, random forest, two types of machines with support vectors and logistic regression). A total of 16,878 records of accidents at construction sites in Australia were used as the initial data for testing the algorithms. The quality of the simulation was checked by six performance indicators. The best values for these indicators were demonstrated by the random forest algorithm. The nature and mechanism of the accident had a significative effect impact on the critical characteristics of the types of injuries.

Thanks to advances in computer vision based on 2D digital images/videos, it has become possible to capture and identify automatically unsafe behavior and risks of personnel in real time. The study of the potential practical application of computer vision in the sphere of the building sector has stimulated a lot of research in this area. The inability of computer vision to accurately detect objects was the main obstacle to its spread in construction practice. Developments in the sphere of deep learning of computer vision, which increase the accuracy, reliability and ability to generalize object detection, have made it possible to eliminate this disadvantage and expand the possibilities of its use on construction sites [137].

Load-bearing buildings (concrete or steel) ensure the stability of engineering constructions due to load redistribution. When performing construction work on the site, people often tend to take a shortcut, crossing the supports to perform their daily tasks and save time. With such unsafe behavior, the probability that a person will be injured or fatally injured increases significantly. To solve this problem of detecting people crossing structural supports during the building of an object, Fang et al. [133] developed an approach to automatic computer vision using a mask region-based convolutional neural network (CNN). The developed algorithms (1) automatically detect the presence of people and (2) confirm their presence, identify the relationship between people and concrete/steel supports. The detection accuracy in the first and second cases was 90.35% and 75.31%. The developed Mask R-CNN can only detect people crossing supports during construction work [134,135,136,139].

Solving safety problems in the building branch determines the increased interest in the improvement and application of computer vision methods. Most of the studies are focused on the recognition of objects and actions, a small part of which studied the recognition of unsafe behavior. Studies developing computer vision-based systems for assessing and predicting safety at construction sites have practically not been conducted [140].

Computer vision techniques can be used to detect and recognize faces at various points to ensure that only qualified people can enter certain areas to perform the necessary work, including working on certain equipment. A motion detection mechanism in certain areas can be implemented by motion detection cameras. The interaction of workers and machines can be recorded in real time, and the emotions of workers can be monitored to check their well-being. The safety of employees in the workplace is always the main concern of companies. Drowsiness detection devices can be installed on cranes to monitor the concentration of crane operators during operation [141]. To minimize the risk of falling from elevations, the presence of safety belts on construction workers was detected using computer vision technologies and convolutional neural networks. The accuracy of such models can reach 99.35% to determine workers who do not have safety equipment to work at heights [126].

The Internet of Things is a concept of network data transmission between physical objects equipped with built-in means and technologies of interaction with each other or with the external environment. This AI technology operating on a building information modeling platform is able to ensure the safety of builders by creating advance warnings and alarms at underground construction sites [4].

Ensuring safety at construction sites with the help of artificial intelligence requires the development and training of algorithms, taking into account the specific tasks expected to be solved on the construction site. The effectiveness of such a tool for ensuring safety at construction sites, is affected both by the reliability and accuracy of AI forecasts and by the amount of funds invested in the development of these technologies.

## 7. Application of AI for Cost Estimation Contracts and Management of Applications, Tenders and Conflicts

In the management of building facilities, AI technologies are an important tool for forecasting the company’s strategy, making bidding decisions, forecasting construction deadlines and computing the cost of the construction process [142]. Digital cost estimation and contract management products are widely used to improve the efficiency of construction project processes at the planning stage (Table 3).

Accurate cost estimation at the preliminary stages of the development of a construction project is crucial for making informed planning decisions. To process the actual data, Dimitriou et al. [153] applied the intelligence of feed forward artificial neural networks (FFANN) to accurately assess the list of volumes of work on the construction of bridges. The FFANN model captures complex relationships in a dataset and provides accurate cost estimates.

Nonparametric estimation of the cost of construction projects using artificial neural networks seems promising for preliminary calculations. The cost predictors characterizing the project/object formed the basis of the estimation concept [154]. The prediction model based on the neural network of back propagation (BP) and improved particle swarm optimization (PSO) is an effective system for managing water consumption costs in construction. The researchers identified four main factors affecting water consumption during construction (the volume of concrete pouring during the day, the weather during the day, the number of workers and the amount of wood used during the day). The forecast data mostly coincided with the actual data, and the relative average error of such a model was 2.66% [155].

The assessment of the consumption of basic materials in civil construction is very important at the initial project stages. Its value is expressed in the influence of the amount of materials on the formation of prices of individual items, and hence on the formation of the total construction cost. Among other things, construction companies use the estimation of the quantity of materials as a basis for offering on the market. The accuracy of the offer taking into account the general conditions of business implementation directly affect the profit that the company can receive from a particular project. Due to the low performance of the initial mathematical model of the pre-cost budget of the installation and construction facility, Lin and Lu proposed a mathematical model of the pre-cost budget of the installation and construction facility based on an improved neural network algorithm. The authors reported that the initial cost of an assembly construction project includes the cost of manufacturing components, the cost of transportation components and the cost of installation components. Based on the cost estimation result, a mathematical model of the pre-cost budget of the object of pre-erected construction is built based on the project parameters [156]. There is not enough available data in the project’s early stages, notably when it comes to data needed to estimate the consumption of materials, and therefore the accuracy of estimating the consumption of materials at the project early stages is lower. The article presents research on the application of AI to estimate the consumption of concrete and reinforcement and the selection of optimal evaluation models. The evaluation model was developed using artificial neural networks. The best model of an artificial neural network showed high accuracy in estimating material consumption expressed as an average absolute error in percentages; 8.56% for estimating concrete consumption and 17.31% for estimating reinforcement consumption [157]. The complexity and interdependence of sources of the risk of delays in the implementation of projects are the main problems of the construction sector. Gondia et al. [158] have developed a machine learning model that is able to accurately predict the risk of delay of construction projects using objective data sources. The prediction was performed on the basis of a Bayesian model.

Since construction contracts usually have an extremely high cost and are financed from the state budget, bidding procedures should not allow inconsistent behavior of bidders. The bidding offer for construction projects includes a number of factors. Minli and Shanshan [159] applied the neural network method to the bidding offer, which not only developed the offer accuracy but also made it more convenient and easier to use. To conduct a transparent bidding procedure, Anysz et al. [142] developed a database that includes hundreds of bidding procedures in the Polish road construction industry based on artificial neural network machine learning methods. As the main factors, the authors indicated the bidding participants, the location of road sections, the cost of applications, the winners and the types of roads. Each procedure was evaluated and assigned to a set with a given level of probability of collusion. Then, two machine learning methods were applied. The first method consisted in training an artificial neural network (ANN) to classify procedures according to the above-mentioned sets. Another method was to use the predictive capabilities of artificial neural networks enriched with fuzzy set theory. The created tool can be used for future bidding procedures as a preliminary check of the appearance of collusion [153].

Minli and Shanshan [159] used a back propagation model in an artificial neural network to predict the bidding offer. The authors identified various factors influencing the bidding offer and applied these factors as input nodes of the network for performing repetitive operations in the network. As a software support tool, the MATLAB application software package was used to reduce the load when writing code and expand the use of a neural network.

To reduce risks and the possibility of managing them, Kim et al. [160] developed a model for predicting financial losses due to accidents, which allows for the organization of effective and sustainable management of construction works. This model was based on the algorithms of deep learning using data on financial losses at construction sites. As a result of validation, the model has increased the reliability of forecasting the cost of financial losses, as well as improved the forecasting method, which makes it suitable for effective management of construction projects and reducing the risk of financial losses.

AI in the field of cost estimation, contract management, conflicts, bids and tenders in construction can significantly improve the efficiency and accuracy of the process, save time and reduce the risk of conflicts. But the lack of a sufficient basis for analysis, for example, emerging conflict situations, leads to distortion of the true picture, incorrect decisions and a devaluation of the obvious benefits of existing technologies.

## 8. AI at the Design Stage

Construction planning is a complex process. Traditional planning methods are usually based on intuition and analysis, which often do not take into account all significant characteristics and are accompanied by errors. As a result, there are project delays, cost overruns and low project productivity [45]. At the design stage, the possibility of using AI technologies is important for predicting the shear strength of the soil, the preliminary cost and the duration of construction. At the initial stage, the volume of such information is limited, but at least the preliminary cost and duration of construction tasks are important for both bidders and customers [161,162]. Similarly, before starting construction, it is important to know the shear strength of the soil in order to have an idea of the ability of the building to withstand the harsh effects of flooding and/or earthquakes.

Artificial neural networks, regression and the support vector machine are the most popular artificial intelligence technologies in the building branch, in descending order. The popularity of these methods is explained by high accuracy, minimal time and easy implementation of the generalized optimal solution [163]. Improving the accuracy of construction planning can be achieved by including additional initial data (historical and site-specific) in the AI analysis, which traditional planning methods often do not take into account. To achieve optimal performance when performing construction projects, Obianyo et al. [45] applied soft computing methods to monitor project activities and evaluate the construction schedule. An ANN and neuro-fuzzy model (ANFIS) was built based on the implementation documents and data from the construction schedule of a residential two-story reinforced concrete frame building. Microsoft Project, MATLAB, and the Levenberg–Marquardt learning algorithm (Trainlm) were used to implement the analysis. MAE, RMSE and R-values (parameters of the loss function) were taken as criteria for evaluating the efficiency of the developed models. The efficiency of the ANFIS model exceeded the ANN model with satisfactory results for solving complex relationships between model variables to obtain an accurate target response.

With the rapid development of Industry 4.0, AI methods made it possible to introduce computer-aided design into the development of building materials [164]. The digital twin contributes to the digitization of building materials. Intelligent application and operation of buildings using the IoT provides the basis for optimal placement of building materials. At the design stage, innovative materials are used in the construction of modern buildings (fiber, carbon fiber, peat blocks, magnesium oxide board, micro cement, infrared heating panels, eco-wool, nanocrete, etc.), the advantages of which are described in [13].

The processes of design and project management in construction are changing significantly with the use of computer vision technologies. These technologies allow for the management of the process of making managerial decisions, and to obtain information, they provide the collection, processing, analysis of digital images, extracting multidimensional data about a real phenomenon [165].

The transition to information modeling began in the last century (USA since the 1970s, the Nordic countries since 2002, China since 2016 and Russia since 2022). In Singapore, more than 80.60% of the building branch has been transferred to BIM technologies, in the USA—at least 50.10%, in the UK—at least 70.00%, in France—at least 60.46%, and in Russia 12.27% of developers use BIM technologies [166].

Object detection using an algorithm of deep learning and computer vision can be used to test components of building materials. This may include contour detection, object counting and object sizing [141]. Cao et al. [167] considered the use of artificial intelligence algorithms in various civil engineering applications, such as forecasting and evaluating various parameters of composite beams and connectors for shear, as well as determining the compressive strength of concrete.

Elhegazy et al. [168] have developed a simple computer model of a multi-storey building ceiling system for optimal planning at the design stage. The proposed AI model is able to predict costs very quickly and accurately when a significant amount of data is available for training the model. In addition, after training the model, deep knowledge of the internal mechanism of AI is not required to apply it in real projects. In such cases, the estimator can simply enter the basic structural properties and the corresponding specific cost of materials in order to obtain accurate output data on the cost of the structural assembly.

Recently, various technologies and systems have been implemented on construction sites to improve communication, coordination, planning and monitoring of the project, including web technologies, cloud computing, building information modeling and tracking technologies. These new applications are commonly used in various technological combinations to improve construction monitoring and allow models to be compared at the project and actual construction stages. Building information modeling is widely used in construction projects to improve communication between different parties at different stages of project design and implementation [169].

Image-based object detection is an important step in monitoring the building progress. High and reliable detection speed can be provided by neural network and machine learning technologies. Since the marking of training data is performed manually, this is a very tedious and time-consuming process.

Braun and Borrmann [170] described a new approach based on 4D information models of buildings in combination with the method of reverse photogrammetry for automatic marking of images of building constructions. A large number of object snapshots are applied to monitor the progress of point cloud processing. Any element of the building model can be projected onto the resulting images by comparing the information model and the resulting point cloud of the building. This approach allows for the automated marking of objects and buildings. For training based on neural network images, these marked-up data are used.

Jiang and Bai [171] determined the location of static vegetation using high-resolution orthoimages obtained using drones. The proposed method includes an ultra-precise neural network encoder based on an orthoimage, a CNN elevation map decoder and an algorithm for disassembling an orthoimage with overlap and assembling an elevation map. The experimental datasets are eight pairs of orthoimages and elevation maps (1536 × 1536 pixels), which are cropped to 64 pairs. This research project expanded the use of unmanned aerial vehicles in construction, made it possible to evaluate the effectiveness of the CNN when shooting sites and strengthened the CNN to work with large-scale images of a construction site. This makes it possible to plan the location of objects in hard-to-reach places at the design stage [163].

When using neural networks in the design of construction projects, it is necessary to take into account the existing risks (insufficient accuracy of predictions due to incorrect training of the model or insufficient initial data; difficulty in evaluating and verifying the design due to the unpredictability of neural network forecasts; information bias or errors; and the limited time required for a complete calculation) when making final conclusions.

## 9. AI in Construction Logistics

Advances in technology, a revolution in business procedures and the need to change warehouse operations as a result of the accumulation of orders, as well as the associated difficulties and lack of managerial skills, paved the way for the emergence of products. Moreover, since warehousing plays a vital role in the supply chain and is a key element of logistics, intelligent product placement is very necessary to improve the management of the organization and achieve success. The use of AI in warehouse operations expands the possibilities of warehouse functioning in logistics, management and coordination [172,173].

The growing fields of application and the depth of autonomous systems in logistics create a new level of tasks for the analysis and development of concepts of human–machine interaction. Due to the shortage of highly qualified personnel in some regions and the goals of increasing efficiency and sustainability, logistics operators must by all means achieve technical progress, including automation of processes [174].

The authors [175] recognize a significant gap in the low interest in logistics 4.0 and AI shown in the research. The concept of logistics 4.0 can be described as big data analytics in real time; for example, the optimization of routing and reduced storage requirements due to new production technologies and autonomous robots with tracking and decision-making systems, leads to optimization of inventory management and information exchange in real time [176]. With the growing popularity of AI technologies, many modern logistics enterprises are trying to use these approaches to optimize logistics communication and improve logistics efficiency [177]. Technological concepts of cyber-physical systems, the Internet of Things (IoT) and the industrial IoT, etc., will allow for the organization of intellectual logistics [178]. Pandian proposed to use AI to optimize work in the warehouse; to automate logistics chains. The author used the algorithms of the IoT, AI and cloud computing to access the resources in the warehouse at any time. The proposed method of automated warehouse logistics uses sensor networks to collect information about the number of goods entering and leaving the warehouse, as well as artificial intelligence to properly handle them in the warehouse, for example, to place them on the right rack, to collect goods from the rack in accordance with the placed order, etc. The information collected by the sensor is transmitted via the Internet to the ThinkSpeak cloud so that customers from anywhere in the world can know about the availability of goods in stock [173].

Arabi and co-authors have developed a way to detect vehicles based on a deep learning algorithm. Taking into account the necessarily small-scale nature of the image datasets of construction machines, the authors presented an improved version of the MobileNet single detector, which is applicable to embedded devices, as a detection model. The results, including a constant average accuracy above 90.24%, confirm the excellent performance of solutions in real time [179]. The Smart Logistics program is aimed at the successful implementation of intelligent and cost-effective supply chains based on flexible and cooperative networks and interconnected organizations. In addition, information exchange is carried out through the use of modern technologies of information and communication, data transmission networks, subjects and sensors, as well as technologies for tracking materials and automatic identification. Automated transport, transition and storage systems supported by autonomous vehicles should provide the possibility of partial and/or complete self-control of systems [178]. Rashid and Louis [180] have shown that automated, reliable, real-time recognition of equipment operation on construction sites can help improve work efficiency, minimize downtime and reduce emissions. Previous efforts to recognize the activity of construction equipment have explored various classification algorithms, accelerometers, and gyroscopes. Such methods require segmentation of continuous operational data with fixed or dynamic windows to extract statistical features. Recent developments in deep neural networks, especially recurrent neural networks (RNNs), open up new possibilities for classifying sequential time series data with repetitive lateral connections. RNNs can automatically learn high-level representative functions through the network, rather than creating them manually; this makes it more suitable for recognizing complex actions. However, the application of RNNs requires a large set of training data, and obtaining this data on real construction sites is a practical problem. The proposed data extension methodology for a deep learning RNN that recognizes the actions of equipment is tested on synthetic data from sample sets that were collected during two real earthmoving operations. The presented deep learning structure surpassed the traditionally used machine learning classification algorithms for recognizing actions in terms of accuracy and generalization of the model [180].

**Figure 4:**
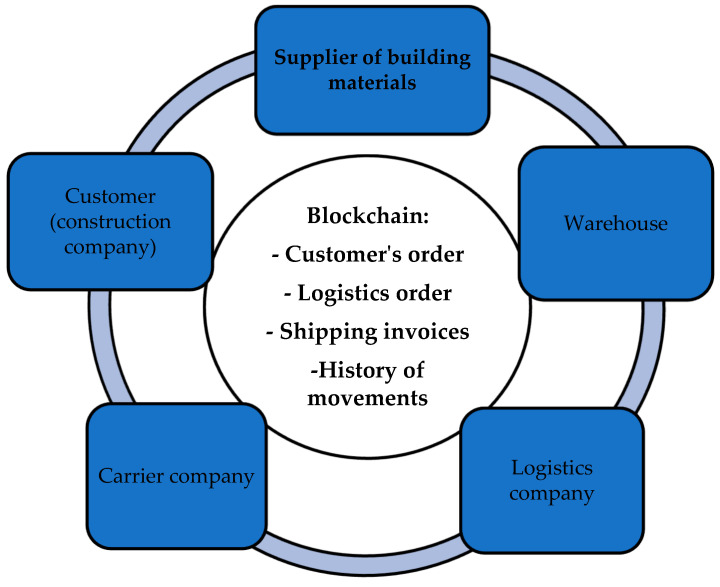
Application of blockchain technology in the building sector (management of supply chains).

The use of blockchain technology (a continuous sequential chain of blocks containing information built according to certain rules) in supply chain management is also gradually expanding. Supply chain management has long sought to reduce costs and improve efficiency; it is also trying to optimize resources and reduce sector fragmentation. Trust has always been an important factor in relationship management and in the effectiveness of supply chain operations. Blockchain technology (Figure 4) helps in solving various tasks of the construction industry (data tracking, contracting and resource transfer in supply chain management). These applications help build trust between partners by making supply chains and other joint project management processes transparent [181].

## 10. AI in the Process of Building Construction

Computer vision methods are promising in the sectors of design, architecture, building and management of facilities to facilitate decision-making processes at the building stage. Manual monitoring on construction sites is extremely tedious and difficult due to the clutter of the sites. Recently, scientists have been interested in the possibilities of using computer vision to solve management tasks on construction sites [182]. With the help of computer vision technologies and other AI methods, Ercan and Wang discovered objects of an automated system for determining the time spent on training construction workers. To obtain a work permit in the construction industry, employees must be trained in technical skills.

To qualify, they must go through a training and evaluation process. During the evaluation, according to the provided technical drawings, students perform assembly (electrical wiring, wall cable duct, etc.). It is a time-consuming process for experts to check (manually and/or visually) the quality and correctness of the installation performed by interns. In order to reduce a significant number of man-hours during the assessment, it is necessary to automate the assessment process, for example, by using computer vision technology [183].

Augmented reality applications allow for a significant increase in the productivity of building work, reducing the number of alterations and improving the transmission of design ideas. Augmented reality can help significantly save time when assembling a pipe coil compared to traditional approaches for both untrained engineers and well-trained professional pipe installers. Those whose cognitive abilities are assessed as low, get more benefit from the augmented reality application than other subjects. Kwiatek and co-authors [184] proposed a manual augmented reality application that was developed to help in the assembly and inspection of pipe assemblies using 3D scanning, scanning and building information modeling, flexible workflow and a touch user interface. The experiments were conducted with 21 professional pipe fitters and 40 engineering students whose spatial cognitive abilities were measured before they assembled a complex pipe coil using traditional and augmented reality tools. The architecture, design, construction and facility management sector is considered one of the most intensive areas where vision-based systems/methods are used to facilitate decision-making processes during the construction phase. Extensive research has been conducted to explore the possibilities of using computer vision to solve management tasks in the field. This article provides an overview of the last decade of computer vision research with an emphasis on modern methods in a typical vision-based scheme and discusses the problems associated with their application.

Statistical analysis of the experimental results for these groups of subjects confirmed several observations: augmented reality can help significantly save time when assembling a pipe coil compared to traditional methods, both for untrained engineers and for well-trained professional pipe installers. Additional studies of augmented reality applications reveal the potential of their impact on the construction sector and expand the range of possible applications [184].

Using images from UAVs Jiang and Bai [163] used a deep learning model to estimate the heights of building constructions. The image-based convolutional neural network encoder, a CNN elevation map decoder and an algorithm for disassembling the image with overlap and assembling the elevation map have compiled the presented methodology. This model allows for the accurate estimation of the height of construction sites using unmanned drones. Li et al. [172] proposed a method based on a deep convolutional neural network algorithm for detecting and classifying building defects during video surveillance inspections. Inspection images collected from sewer lines (24.7 km) were used to train and test the neural network. The hierarchical approach to classification has increased the accuracy of detecting high-level defects by almost 5.00% (from 78.40% to 83.22%).

Zhang et al. [136] used digital twin technology to assess the movement of workers and construction equipment on the construction site. To do this, the authors used an open-source video dataset of 2064 video clips with 5 types of actions for excavators and dump trucks, including the corresponding optical flow dataset for predicting movements. To support intelligent construction, a digital twin is used, which has become a universally recognized concept of the virtual representation of a physical object. The study says that recognition of the movement of construction equipment and the actions of people on virtual construction sites is no less important. A similar system is being developed in Kuzbass (Russia) within the framework of the project “Geoinformation system of digital regional management” [185]. Comparing a significant number of studies on the recognition of human actions, the results of which can be extended to builders and other construction workers, the number of studies on the recognition of the actions of construction equipment is limited due to the insufficiency of video datasets with the actions of construction equipment for training.

However, such a dataset is still relatively insufficient compared to the datasets available in other areas of applications using deep learning solutions. It is expected that a higher level of accuracy will be achieved after a much larger dataset is developed. It is important to develop more complete sets of video data for different types of equipment in different conditions, for example, when the camera is moving or in adverse weather conditions (for example, in strong wind or rain). In addition, it is important to study the perception of actions in which several types of construction equipment move simultaneously. In many cases, a surveillance camera captures a construction site with several pieces of equipment; this becomes a problem when different types of equipment require different action recognition algorithms in one frame to achieve better recognition performance.

Creating a digital twin of a construction object based on huge amounts of data about a building or an engineering object provides an idea of the performance and behavior on the construction site in real time, providing intelligent information exchange from a real object to its digital twin and optimizing the performance of the control tool. Advanced analytics in real time is carried out in a digital double, and adjustments to the construction object are carried out by a human operator, software or robots [186].

Artificial intelligence systems will potentially be able to study very accurate and detailed models of individual objects and make a competent choice of management. The industry is already using digital twins to optimize equipment maintenance, identify possible failure options for parts and automate more processes. The capabilities of digital twins will only expand thanks to more advanced sensors, faster cloud and advanced computing, and constantly improving AI [187].

Despite the historically slow introduction of new technologies, construction companies are beginning to successfully use AI technologies for everything (from task automation to data mining to obtain useful information). Options for using AI technologies when performing work on real construction sites are given in Table 4. Here are a few ways AI is being exploited in the building branch today:

China and the USA are the undoubted leaders in the introduction of AI technologies in different areas, including construction [207]. To form a new infrastructure, China has already developed computing centers, deep artificial intelligence models and learning platforms, thus, providing digital transformation in various areas of the real economy (vehicles, manufacturing, building branch, etc.). Sufficient development of robotic technologies with the support of artificial intelligence technologies expands the possibilities of their application in construction, mining, disaster response and in types of work that require a large amount of labor. The US surpasses China in terms of academic infrastructure, without which the development of the necessary technologies slows down. The Beijing Academy of Artificial Intelligence has already been established in China, and leading universities are opening training courses in AI technologies.

By 2030, China will try to become a global innovation center in the area of AI. According to Roberts et al. [208] by this time, the growth of the main artificial intelligence industry will amount to CNY 1 trillion again with the necessary development of an appropriate legislative framework. According to the Chinese Academy of Sciences, artificial intelligence is used to monitor the behavior of workers on building sites throughout China [209]. However, in 2021, the share of North American AI in construction accounted for the largest market volume, which is provided by government initiatives in the field of AI in the construction sector, a sufficient population with high purchasing power and constant investments in automation [210].

The use of AI in logistics at a construction site can be useful for forecasting resource needs, planning and optimizing delivery routes, automatic inventory management, improving the efficiency of material and technical resource management processes, allocating resources and manpower to increase productivity and reduce costs, detecting trends, anomalies and problems and making recommendations for improving processes and the results of the work. But at the same time, it is necessary to integrate AI with various systems and technologies, train personnel to work with it, ensure data security and protect personal information, and all this requires significant organizational and financial costs.

## 11. Advantages and Disadvantages of AI in Construction

In the near future, AI and machine learning technologies will play a major role in improving the quality of construction projects and the development of intelligent construction in general [211]. However, these technologies have both advantages and limitations.

The use of innovative and breakthrough business models, in particular AI methods and technologies adapted to collect data from construction sites and for existing construction applications, will allow for the support of existing construction companies and this economic system in general. There is still no implementation of the platform approach in the entire industry, but its partial implementation already shows great advantages. Artificial intelligence algorithms will allow the construction data to be structured and linked to the data of other organizations, guaranteeing the technological and legal security of users. The combined database will allow the use of various programs and applications in the building branch [212]. However, when implementing AI methods in construction projects, there are disadvantages, such as corruption, fragmentation of buildings, inappropriate risk allocation, inefficient design, limited standardization, insufficient planning time and delayed implementation of AI in the field of management and execution [213].

In the modern world, AI is rapidly changing the rules of the game in various industries, including the construction segment. Combining AI technologies with the development of computing resources will not only optimize processes and stimulate innovation but also make them available everywhere, on many construction sites. For specialists in the field of construction, it is important to understand all the advantages and disadvantages of using AI in order to make informed decisions, overcome emerging difficulties and ensure the economic efficiency of their company [214]. Companies can strategically implement AI technologies, fully understanding the potential benefits (increased efficiency and security, improved project planning, etc.) [70]. Khanzode and Sarode [215] and other researchers [65,216,217,218] identified the main advantages and disadvantages of AI methods (Table 5).

Since AI-based programs and applications are susceptible to attacks by hackers and cybercriminals, they lead to huge consequences for time, quality and cost. Most significantly, the safety of builders can be jeopardized, which can lead to loss of life or life-threatening accidents. For instance, a computer vision system that recognizes automated building equipment can be hacked, which can lead to accidents. At this moment, there is a global shortage of personnel of the highest category, in particular, AI engineers who have the necessary skills to lead serious developments in various industries. Also, investing in AI-based equipment requires high costs. The cost of AI-based equipment maintenance still remains high [4]. Forecasting of purchases and supplies of material and technical resources for the construction of various kinds of facilities is not recommended without the use of AI methods. The successful solution of a number of tasks of construction projects is guaranteed by the application of AI methods, which iterates through numerous scenarios in a limited time and is able to learn from calculated algorithms:

- The analysis and identification of the necessary stock of material values and the turnover of inventory [214] determine the profitability of the construction business;
- The automation and aggregation of document flow, improvement of logistics management by reducing the number of dealers and direct access to resource suppliers [219];
- Forecasting the demand for material resources and the formation of a supply schedule [220].

## 12. Prospects and Limitations of AI Use in Construction

Despite the significant successes of AI in various fields, it still faces limitations in understanding and performing complex tasks that require human judgment, creativity and intuition. As a result, AI will not be able to completely replace the human factor in all aspects of construction projects. There are several restrictions on the realization of AI in construction [221]:

- Algorithms require sufficiently large amounts of data for accurate training (training the system to detect a worker without a helmet required machine learning based on millions of different images of a worker in a helmet), and insufficient data volume excludes the possibility of conducting such training [221];
- The need to integrate all possible sources of information and provide conditions (a single platform) for storing, accessing and processing the collected data [221];
- Most construction organizations do not have enough data to train their algorithms [222];
- The high level of requirements of data processing specialists can be met only by large players in the construction business [223];
- For the proper functioning of AI technologies, human service is necessary (each event or decision depends on several external factors leading to various consequences) [221].

In comparison with other industrial sectors, the digital transformation of processes in the construction sector is significantly lagging behind [224]. The use of AI technologies and the development of machine learning algorithms make it possible to optimize construction processes at all stages of the construction of various objects [225]. Analysis of the condition of the building branch in recent years shows that there is a growth in the costs of developing and implementing AI algorithms in construction [226]. The accumulation of necessary data occurs unevenly, at different stages of the construction project implementation [227]. The BigData necessary for training AI neural networks are formed from video materials from various construction sites, information and data obtained from security sensors, digital models of buildings, etc. [228,229].

AI technologies and ML algorithms in the building branch have almost unlimited application potential [24]. AI can maintain a high speed of complex decision making; therefore, there will be a shift towards the algorithms of empowerment in the implementation of construction projects [230]. The introduction of design systems, the use of autonomous construction equipment and robots in combination with the accumulation of significant amounts of data for training neural networks will increase the efficiency of the implementation of construction production facilities [231]. Artificial intelligence will change the models of project implementation in the building branch and replace the human workforce, reducing workplace injuries and increasing the efficiency of operations [232]. Artificial intelligence technologies, the Internet of Things and robotics can potentially reduce construction costs by up to 20.42%. Cameras attached to robots collect information; in modern buildings, artificial intelligence plans plumbing and electrical systems. To prevent errors during construction, performance and safety problems, the AI systems monitor the interaction of workers, equipment and objects on the building site in real-time [233]. At all stages of strategic construction planning, it is possible to benefit from the use of machine learning algorithms, for which there are practically no obstacles to application in the building branch [234]. It is important to note that the issue of ethical and social aspects of AI implementation for society and employees of the industry deserves special attention [230].

The future of AI in the building sector, and the industry as a whole, is promising. Technologies and computing power continue to develop, and it is obvious that AI is likely to become an integral part of the construction process in the near future [235]. Companies that are ready to adapt to these changes and invest in AI technologies in a timely manner will have the opportunity to benefit and maintain a competitive advantage in the industry [236].

According to Allied Market Research [68], the cost of construction management software will reach RUB 2.3 billion by 2028. In the long term, the introduction of AI technologies in construction will depend on the industry’s ability to apply innovative approaches to project management during their implementation, a conscious comparison of advantages and disadvantages, and a willingness to invest in the development of the workforce. The capabilities of artificial intelligence will transform the industry and make the emerging artificial environment a common phenomenon [237].

AI methods and technologies can significantly improve work on construction sites, providing new opportunities and increasing efficiency in various aspects of construction activities. Currently, AI technologies support machines that have much higher computational abilities than humans, for example, the ability to sift through huge amounts of data and use this data to make better decisions. AI allows computers to adapt to new data—basically, learning without explicit programming. AI can analyze large amounts of data and take into account numerous factors (weather, time schedule, budget, etc.) to predict potential problems and optimize the construction process. AI allows for the creation of automatic control systems that can control and regulate various aspects of construction. Robotic systems can perform repetitive tasks (laying bricks and installation of steel structures), while maintaining the speed and quality of the work performed. Computer vision can be used to automatically scan and analyze surfaces, identifying defects, cracks or other problems. Smart building systems and AI can automatically adjust lighting, heating and air conditioning to reduce energy costs and increase comfort and thereby optimize energy consumption at the construction site. To ensure security management, video surveillance systems based on AI technologies can automatically detect potentially dangerous situations (workers falling, lack of protective equipment, etc.) and warn about them.

Additional bonuses appear when various artificial intelligence approaches interact in one construction project, complementing each other. In construction, machine learning can be used both for analyzing large amounts of data, pattern recognition, forecasting the cost and duration of projects, and for automating decision-making processes. Robotics at a construction site allow the use of autonomous robots that are programmed to perform operations such as laying bricks or plastering walls, dismantling, lifting and moving heavy materials, etc. Computer vision can simultaneously be used to control the quality of work, monitor safety on the construction site, detect defects in building materials, etc. Knowledge-based design uses knowledge bases and expert systems, previously accumulated knowledge and experience to create automated design processes and decision making in construction, as well as to reduce time and improve the quality of the design.

In general, these different AI approaches in construction complement each other, allowing automation and optimization of various aspects of construction activities to improve efficiency, safety and quality of work. Obviously, regardless of the industry, the potential of AI is huge; with a competent combination of data analysis, machine learning and other AI technologies it can increase efficiency, reduce costs and optimize any working environment. Thanks to the ability of AI to analyze data, learn and make predictions, the future possibilities of its application at construction sites will only increase.

## 13. Consequences of the Use of Artificial Intelligence in Politics

Over the past few years, there has been significant progress in the field of AI in areas such as smart vehicles and smart buildings, medical robots, communications and intelligent education systems. It is expected that these achievements will have consequences in several areas of politics and government (economic, social, educational, etc.), and increasing attention is being paid to it around the world [88].

In the documents, both the US and the UK, it is noted that when considering approaches to AI regulation, attention should be paid to ensuring that such approaches do not hinder innovation and progress. Moreover, the UK report calls for the creation of an AI Commission, which will be tasked with determining the principles of AI development and application, providing recommendations to the government and promoting public dialogue [238]. The draft report of the European Parliament also recognizes that existing legal regimes and doctrines can be applied to robotics but notes that “the current legal framework will not be enough to cover the damage caused by new generation robots.” Therefore, it requires the adoption of the EU Directive on the rules of civil law in relation to robotics as well as the guiding ethical framework for the design, production and use of robots [88]. It is also proposed to create a European Agency for Robotics and AI to provide technical, ethical and regulatory knowledge to support the EU and its member states [93].

The political implications of AI also attract the attention of intergovernmental organizations such as the United Nations, the G7 and the Organization for Economic Cooperation and Development. Governments increasingly believe that AI will benefit from international cooperation in promoting research and development as well as in identifying appropriate responses to relevant problems [239]. In the United States, the government is encouraged to develop an AI-related international engagement strategy and collaborate with other stakeholders in developing AI standards. The draft report of the European Parliament calls for international harmonization of technical standards, mainly in order to avoid risks of market fragmentation and to uniformly solve consumer problems. It also encourages international cooperation in setting regulatory standards under the auspices of the UN [240].

However, markers of the relative danger of uncontrolled AI technologies require protective legal and policy measures. In September 2017, Oren Etzioni, CEO of the Allen Institute for Artificial Intelligence, recommended that regulators focus on the “tangible impact” of AI, rather than “defining” or “harnessing” the “amorphous” field. At the same time, as the field and risks of AI materialize, legislators and politicians are shifting towards how to balance the risks of national security with the promotion of technological advances and access to information [241]. The EU Law on Artificial Intelligence regulates specific applications such as law enforcement, voting and social networks. China, Brazil and other countries apply their own approaches to regulation [241].

Meanwhile, US Congressional leaders dealing with AI technologies suggest it could be months before a comprehensive federal law is passed to supplement their meager existing regulations. The John S. McCain National Defense Authorization Act of 2019 struggles to define AI. The 2020 National AI Initiative Act largely instructs the President and federal agencies to assess the potential and risks of artificial intelligence [242].

Taken together, legal maneuvers to regulate AI entail many constitutional and other legal problems concerning jurisdiction, powers, business interests, civil liberties and individual rights. The wave of upcoming lawsuits has already begun with numerous class-action lawsuits against industry leaders on the grounds that their chatbots improperly acquired copyrighted data on the Internet. In June 2023, a public figure in Georgia stated that ChatGPT had provided defamatory information suggesting that he had embezzled money [243].

New legal efforts to respond positively to AI-related public health and safety risks are centered around international agreements based on existing governance systems that comply with humanitarian law. Stakeholders are calling for the creation of broad coalitions from several countries that manage “democracy-affirming” technologies that promote human rights and fight authoritarianism. US diplomatic agreements with the EU, Israel, India and other jurisdictions are under negotiation. It is hoped that a global consensus on AI control can be achieved through these and other alliances [244]. All political consequences should be taken into account and weighed when developing and implementing the use of AI in order to mitigate its negative consequences and transform it into a positive tool for society.

## 14. Conclusions

AI methods are of great importance for the development of the construction industry. Their application has the potential to organize a more reliable, automated, intensive and cost-effective construction process. The construction industry is facing a productivity challenge and countless other challenges that could potentially be solved with AI. With the increase in the amount of data generated throughout the life cycle of a building and the advent of other digital technologies, AI has the opportunity to use this data and the capabilities of other technologies to improve construction processes. To answer the research questions in this work, the extent to which artificial intelligence technologies are used in construction was studied. Concepts, types, components and subfields of AI are briefly explained, as well as works using these subfields. The basic information on the areas of application, advantages, limitations and benefits of each subdomain of AI used in construction was presented.

This study shows that although some artificial intelligence technologies have been used in construction research, recent advances in these technologies have improved significantly, and the introduction of new, more powerful AI technologies is relatively slow. Moreover, this study identified and discussed some additional opportunities and open research challenges for AI research in construction. Although the application of AI is gradually increasing, its relevance is further enhanced by other new trends, such as BIM, IoT, quantum computing, augmented reality, cybersecurity and blockchain. We investigated some of the problems affecting the implementation of AI in the industry and solved them with the help of recommendations. This study is a useful source of information for researchers and practitioners about relevant AI applications and research in the construction industry. This study presented a clear understanding of internal capabilities as well as potential obstacles in application areas. Construction stakeholders such as regulators, decision makers, highly skilled workers and digital enthusiasts can use the information provided in this study to clearly identify the path to AI adoption and reduce the potential risks associated with it.

The development of the construction industry is primarily associated with solutions based on AI technologies. At the same time, a small amount of machine-readable documentation and a low percentage of the use of BIM modeling technologies remain a limiting factor. There are opinions that there are two areas where AI can be used: integrated development of territories (with the help of AI solutions, it is much faster and cheaper to search, select and analyze territories to involve them in the process of integrated development) and the use of AI in expert work (AI algorithms are gradually replacing experts when analyzing the results of engineering surveys, project documentation, dataset, machine processing and data indexing). However, if efficiency increases by reducing labor costs, then it is necessary to solve the ethical issues that arise as well as the issue of retraining people. Another solution implemented in construction using AI is to monitor project financing and inform banks about facilities for which construction progress requires attention or verification. In general, the use of AI technologies in construction can improve processes, increase productivity, reduce costs and improve safety. This can be especially useful in modern conditions when construction is becoming more complex and requires more attention to detail.

## Figures and Tables

**Table 1:** Distribution of AI technology applications in the building sector.

Application Areas	AI Technologies					
	A	B	C	D	E	F
Health/Safety	[77,78,79]	[80,81]	-	-	[82,83]	-
Project planning and design	[83,84,85]	[86,87]	[88,89]	[4]	[90,91]	[84,92]
Cost estimation	-	-	[4,93]	-	[94,95]	[96,97]
Contracts, conflict management and tenders	[98]	[99,100]	-	-	[101]	[102]
Supply management, equipment and logistics	[2,4,103]	-	[104]	-	[13,105]	[45]
Site monitoring and performance evaluation	[106,107]	[108,109,110]	-	[111,112]	[113]	-
Risk management	[114,115]	-	-	[4,99]	[116,117]	-
Stability	-	-	-	-	[4,86]	[118]

A—Machine learning; B—computer vision; C—automated planning; D—robotics; E—knowledge-based systems; and F—optimization.

**Table 2:** AI approaches used to ensure safety in construction.

The Proposed Safety Solution	AI Method	Prediction Accuracy, %	Sources
Early detection and accident prevention platform	Computer vision and AI	97.40	[121]
Predicting accidents at a construction site	Artificial neural network and case-based reasoning	86.00	[127]
Detection of abnormal situations and transfer of information to the operator	Machine learning and decision tree	-	[128]
Road construction safety monitoring platform	AI and UAVs with deep learning algorithms, including YOLOv4 and DeepSORT	-	[128]
Practical guide to the preliminary prevention of accidents on construction sites	Artificial neural networks	80.00	[129]
Detection of hazards at construction sites	Convolutional neural networks based on regions	-	[130]
Assessment of safe and unsafe conditions on construction sites	Building information modeling and computer vision	-	[128]
Model for determining potential risks on construction sites (fatal accidents)	Machine learning methods: (AdaBoost analysis, decision tree and logistic regression, random forest)	91.98	[131]
Helmet wearing recognition of construction workers	Convolutional neural network	83.89	[132]
Proximity warning alert system	Automatic computer vision, which uses a convolutional neural network model based on regions	90.00	[133]
Safety belt detection system for workers to reduce the risk of falling from elevations	Computer sight and convolutional neural networks	99.0	[134]
A system that ensures the safety of builders by creating advance warnings and alarms at underground construction sites	Internet of Things	-	[4]

**Table 3:** Artificial intelligence approaches used to estimate costs and manage contracts and conflicts, bids and tenders.

The Proposed Safety Solution	AI Method	Sources
Estimation and planning	BIM-IoT-blockchain technology; BIM for estimation of the time and cost; Deep learning for estimation of the time and cost.	[143,144,145]
Supply chain management	Blockchain technologies; Risk monitoring system; Mobile supply chain.	[2,146,147,148]
Construction contract management	Machine learning algorithms; Blockchain technologies.	[103,149,150]
Cost management	Blockchain technologies and encryption.	[4,151]
Audit systems of financial statements of building company	Statistical and cluster analysis; Blockchain technologies for audit of the BIM modification.	[4,88,151,152]

**Table 4:** Construction objects using AI technologies.

Object	Technologies Used	Country	Sources
Dam construction	AI technologies and 3D printer	China	[188]
Safety monitoring at construction sites	Internet of Things and remote sensing	China, USA, Russia, Canada, etc.	[136,189,190,191,192,193,194]
Effective construction management	ESKIMO system	Germany	[195]
Construction of a two-storey building	3D printer	India (Larsen & Toubro Company)	[196]
Construction management	Digital twins created from images of robots with cameras and neural networks	USA (Droxel company)	[197]
Design of buildings	BIM technologies	Great Britain, Russia, France, etc.	[136,198,199,200]
Helsinki Airport and Tampere Deck and Arena	BIM technologies	Finland	[136]
Reinforced concrete bridge	BIM technologies	Peru and France	[136]
Pidekso Dam	BIM technologies	Indonesia	[136]
Motorway with tunnels	BIM technologies	Norway	[136]
Auckland International Airport	BIM technologies	New Zealand	[199]
Shanghai Tower, Disneyland and Shanghai World Financial Center	BIM technologies	China	[199,201]
City of New Alamein	BIM technologies	Egypt	[201]
Metro	Autocad civil 3D and Autodesk Revit software, Synchro Pro and BIM technologies	Dubai (Acciona Company)	[202]
Road construction	Digital twins and shooting from a UAV	Germany, China, Czech Republic, Italy, USA, Malaysia, etc.	[203,204,205,206]

**Table 5:** Advantages and disadvantages of using AI in construction.

Advantages	Disadvantages
Task completion speed	High costs at the investment stage
Safe, efficient and timely execution of projects	Technology costs
Ability to solve complex and stressful tasks	Expensive and complex equipment repairs
Possibility of simultaneous execution of several processes	Risks of unemployment of specialists
Reduction in administrative processes	The need for training and retraining of the workforce
Reducing the cost of project implementation	Efforts and funds for the maintenance of AI technologies
Forecasting of safety and quality risks at work sites	Risks of hacker attacks and data leaks
Minimum number of errors	Computational constraints
The possibility of long-term forecasting of events	Presence of errors
Ability to process qualitative and quantitative factors	The operation and efficiency of the system depends on an AI specialist
Reduction in operating costs	Increasing technological dependence
Reduction in paperwork	Dependence on electricity and internet connection
No need for control (operation without an operator)	Limitations of the scope of application

## Data Availability

Data are contained within the article.
